# The Identification of Gyrophoric Acid, a Phytochemical Derived from Lichen, as a Potent Inhibitor for Aggregation of Amyloid Beta Peptide: In Silico and Biochemical Evaluation

**DOI:** 10.3390/ijms26178500

**Published:** 2025-09-01

**Authors:** Meixia Yang, Haitao Hu, Jin Gao, Queenie Wing Sze Lai, Farkhod Eshboev, Ka Wing Leung, Tina Tingxia Dong, Qin Xu, Karl Wah Keung Tsim

**Affiliations:** 1 Division of Life Science and Center for Chinese Medicine, The Hong Kong University of Science and Technology, Hong Kong SAR, China; mxyang@ust.hk (M.Y.); gaojin@xzhmu.edu.cn (J.G.); queenielai@ust.hk (Q.W.S.L.); eshboevf@ust.hk (F.E.); lkwing@ust.hk (K.W.L.); botina@ust.hk (T.T.D.); 2 Shenzhen Key Laboratory of Edible and Medicinal Bioresources, HKUST Shenzhen Research Institute, Shenzhen 518057, China; 3 Department of Physics, The Hong Kong University of Science and Technology, Hong Kong SAR, China; hhuar@connect.ust.hk (H.H.); qinxu@ust.hk (Q.X.); 4 Department of Neurobiology and Cell Biology, Xuzhou Key Laboratory of Neurobiology, Xuzhou Medical University, Xuzhou 221004, China; 5 Institute for Advanced Studies, New Uzbekistan University, Tashkent 100007, Uzbekistan; 6 S.Yu. Yunusov Institute of the Chemistry of Plant Substances, Academy of Sciences of Uzbekistan, Tashkent 100170, Uzbekistan

**Keywords:** Alzheimer’s disease, gyrophoric acid, natural bioactive compound, amyloid beta peptide, fibril disassembly, molecular dynamics, therapeutic development

## Abstract

Alzheimer’s disease (AD) is characterized by amyloid-beta (Aβ) plaque accumulation and neurodegeneration. This study identified gyrophoric acid, a lichen-derived phenolic metabolite, as a dual-action Aβ42 inhibitor preventing aggregation and disassembling of mature Aβ42 fibrils. Integrated in silico studies revealed that gyrophoric acid was a strong thermodynamic stabilizer of Aβ42 (MM–GBSA: −27.3 kcal/mol) via entropically driven hydrophobic interactions and disruption of aggregation-prone conformations (100 ns MD simulations). Through biochemical analysis of the fluorescent dye thioflavin T (ThT), gyrophoric acid induced rapid Aβ42 fibril disassembly within 5 h, with time-lapse confocal microscopy quantitatively confirming the near-complete dissolution of large aggregates by 24 h. ADMET profiling revealed favorable pharmacokinetics (moderate oral absorption: 48.5–57.3%; low toxicity) and Lipinski’s rule compliance. These results establish gyrophoric acid as a promising natural bioactive compound for anti-AD therapeutics with a unique hydrophobic-stabilization mechanism.

## 1. Introduction

Alzheimer’s disease (AD), a progressive neurodegenerative disorder, manifests through irreversible cognitive decline, memory loss, and neuropsychiatric disturbances, ultimately leading to dementia [1,2]. Pathologically, AD is defined by extracellular amyloid-beta (Aβ) plaques, intra-neuronal neurofibrillary tangles of hyperphosphorylated tau proteins, synaptic degeneration, and cholinergic dysfunction [3,4]. Despite decades of research, existing therapies, i.e., primarily targeting Aβ aggregation or acetylcholinesterase (AChE) inhibition, offer only symptomatic relief and fail to halt disease progression [5]. With global AD cases projected to triple by 2050 due to aging populations and the absence of disease-modifying treatments, innovative strategies addressing its multifactorial pathogenesis are imperative [6,7].

The central etiologies of AD are oxidative stress, Aβ fibrillization, and tau hyperphosphorylation, which synergistically drive neuronal loss and synaptic failure [8,9]. From the viewpoint of modeling methodology, Aβ can be safely considered a robust intrinsically disordered protein (IDP) [10]. Global conformational rearrangement and the formation of intermolecular secondary beta-sheet structures are caused by fibrillization, and the secondary structure of Aβ peptide is a highly toxic synthetic derivative and structurally stable [11]. Aβ aggregation remains a pivotal therapeutic target, as soluble oligomers and mature fibrils disrupt membrane integrity, impair mitochondrial function, and propagate neurotoxicity [4]. Natural products, with their structural diversity, multitarget potential, and favorable safety profiles, have emerged as promising candidates in modulating Aβ pathology [12]. In particular, gyrophoric acid, a lichen-derived secondary metabolites belonging to the depside class of polyphenolic compounds [13], has shown remarkable antioxidant properties [14,15] and anti-amyloid activity [16]. Our prior work demonstrated that application of gyrophoric acid effectively suppressed Aβ42 fibrillation, destabilized preformed fibrils, and protected neuronal cells from Aβ42-induced cytotoxicity, highlighting its neuroprotective potential [16].

Translating natural compounds, e.g., gyrophoric acid, into clinical use requires overcoming challenges related to pharmacokinetic optimization and target specificity. For instance, gyrophoric acid’s hydrophobic structure facilitates interactions with Aβ42’s amyloidogenic regions, but it may also limit its solubility and blood–brain barrier (BBB) permeability. To address these limitations, we employed an in silico pipeline integrating molecular docking, molecular dynamic (MD) simulations [17], and ADMET (absorption, distribution, metabolism, excretion, toxicity) profiling [18,19] to reveal the potential of gyrophoric acid as a drug. A number of experimental fibril structures of Aβ have been reported recently [20,21]. The importance of pentamer and hexamer oligomers in Aβ aggregation has been highlighted in a number of studies [22]. Aβ17–42 pentamer and hexamer oligomers, called paranuclei, are reported as potent on-pathway intermediates that are converted into U-shape fibrillar structures [22]. The pentameric model 2BEG has been used as a suitable model in a number of recent computational studies [20,23,24]. Thus, a pentameric model based on a solid-state NMR Aβ42 protofibril structure (PDB ID: 2BEG) was selected for MD simulations in the present study. In addition, in vitro biochemical analyses using thioflavin T (ThT) assays and real-time confocal microscopy further confirmed the role of gyrophoric acid in Aβ42 fibrillation. The current findings not only uncover the entropic dominance of gyrophoric acid’s Aβ42 stabilization in mirroring the “conformational selection” mechanism of intrinsically disordered proteins, but also lay the groundwork in developing next-generation anti-amyloid therapeutics with improved bioavailability and target engagement.

## 2. Results

### 2.1. XP Molecular Docking and MM–GBSA Calculation

Ramachandran plot analysis (Supplementary Figure S1) demonstrated high stereochemical validity of the Aβ42 structure (PDB ID: 2BEG), with >90% of residues residing in favored (allowed, red) and allowed (marginally allowed, yellow) regions. Less than 10% of residues occupied disallowed regions (white areas, marked as red dots), confirming exceptional structural reliability. Outlier residues localized predominantly to flexible protein segments and showed no significant impact on the molecular docking. To validate our docking protocol, we performed control docking using an Aβ42 structure (PDB ID: 2BEG) containing co-crystallized curcumin, confirming the protocol reliability (see Supplementary Figure S2). Comprehensive analysis of XP docking and MM–GBSA results showed that curcumin exhibited MM–GBSA binding free energy of −30.57 kcal/mol with the amyloid beta peptide (Supplementary Table S1). This value indicated the high stability of the curcumin–peptide complex.

**Figure 1:**
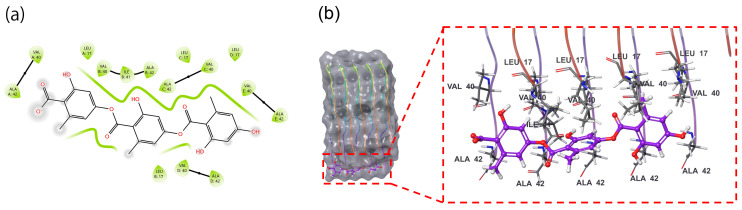
(**a**) High-accuracy XP 2D molecular docking (Schrödinger Maestro 13.5 software) of gyrophoric acid into the active site of Aβ peptide. Binding free energy of −27.3 kcal/mol indicate that gyrophoric acid binding to Aβ peptide is relatively stable. (**b**) 3D binding mode of gyrophoric acid into the active site of Aβ peptide. Gyrophoric acid binds to the surface of the active pocket of Aβ peptide. Residues Ala42 and Val40 on chain E, Val40 and Ala42 on chain C, and Ala42 on chain B contribute to the hydrophobic interactions with gyrophoric acid. Gyrophoric acid interacts with Aβ peptide through non-covalent bonds.

Computational profiling of gyrophoric acid revealed a paradoxical binding signature: the MM–GBSA-derived binding free energy (dG bind −27.3 kcal/mol) indicated robust thermodynamic stabilization of the complex (Supplementary Table S1). Gyrophoric acid’s affinity for MM–GBSA dG-binding energy is competitive with clinically studied natural products, e.g., curcumin, diltiazem, rosiglitazone, milnacipran, and dapsone [25]. Structural representation of gyrophoric acid showed possible binding to the surface of Aβ42 peptide’s active pocket. Despite the spatial proximity, no significant non-covalent interactions, e.g., hydrogen bonds, hydrophobic contacts, or π–π stacking, were identified between gyrophoric acid and the protein. This dichotomy implied a multiphase binding mechanism (Figure 1a), where kinetic barriers during the initial docking phase, such as suboptimal ligand positioning or partial desolation penalties, limited the binding efficiency. Hydrophobic interactions between gyrophoric acid and key residues of Aβ42 peptide were identified (Figure 1b). Critical residues mediating the hydrophobic contacts included Ala42 (chain E), Val40 (chain E), Val40 (chain C), Ala42 (chain C), and Ala42 (chain B). These residues collectively formed a hydrophobic pocket that stabilized gyrophoric acid binding, aligning with the strongly favorable MM–GBSA binding free energy (dG bind −27.3 kcal/mol). The absence of conventional hydrogen bonds, or electrostatic interactions, suggests a dominance of entropically driven hydrophobic effects in the ligand stabilization.

This behavior mirrors the “conformational selection” paradigm observed in intrinsically disordered proteins like Aβ, where ligand binding stabilizes transient low-population states of the peptide [17,26]. Notably, the absence of canonical hydrogen bonds and the dominance of hydrophobic contacts suggest that the entropic gains from solvent displacement and van der Waals packing, rather than the enthalpic forces, drive the observed stabilization (Figure 1). These findings raise intriguing pharmacological implications, i.e., the weak initial binding may reduce off-target interactions, while strong thermodynamic stabilization could enhance target residence time.

### 2.2. Molecular Dynamic (MD) Simulation

**Figure 2:**
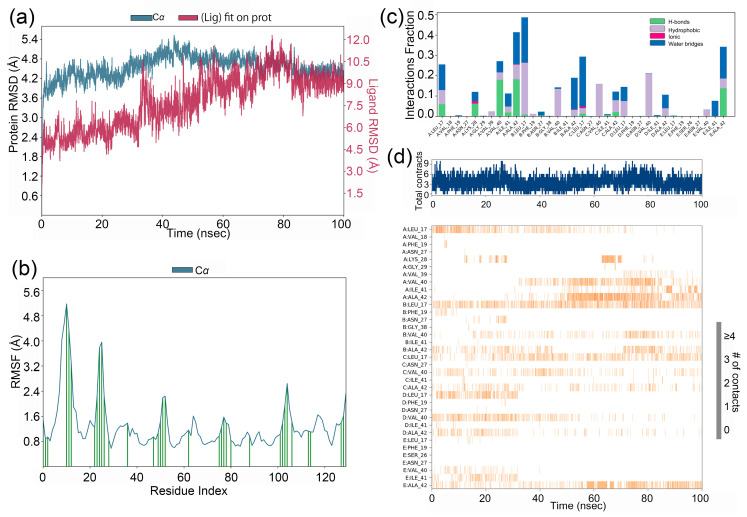
(**a**) RMSD values for gyrophoric acid–Aβ peptide complex during MD simulation. The gyrophoric acid–Aβ complex remains relatively stable after 70 ns of molecular dynamic trajectory. (**b**) RMSF plot for Cα of Aβ peptide chain residues with gyrophoric acid. Protein residues that interact with the ligand are marked with vertical green bars. (**c**) Gyrophoric acid–Aβ peptide interactions during MD simulation. Protein–ligand interactions were monitored throughout the simulation and categorized into four types: hydrogen bonds, hydrophobic contacts, ionic bridges, and water bridges. The key amino acid residues contributing to the binding of gyrophoric acid to the Aβ peptide protein included chain A Leu17, chain A Val40, chain A Ala42, chain B Leu17, chain C Leu17, and chain E Ala42. The dominant interactions observed were water bridges, hydrophobic contacts, and hydrogen bonds. (**d**) Time-dependent interactions between gyrophoric acid and specific amino acid residues of Aβ peptide across the simulation trajectory. Changes over time in the interactions between gyrophoric acid and specific amino acids of Aβ. The amino acid residues Leu17, Val40, and Ala42 on chain A, Leu17 on chain B, Leu17 on chain C, and Ala42 on chain E have multiple contacts with the ligand (represented by darker orange coloration).

Molecular dynamic (MD) simulations, offering atomic-level precision and high temporal resolution, are now integral to drug development and drive the design/optimization of therapeutics (e.g., small molecules, peptides, proteins) and elucidate the structural basis of diseases. MD simulations of gyrophoric acid with the Aβ42 peptide complex were performed for 100 ns, and the resulting trajectories were analyzed for conformational stability (Figure 2a). As shown in the figure, the root-mean-square deviation (RMSD) plotted against simulation time revealed minor fluctuations (≤2.0 Å) across all systems, indicating that the complexes achieved stable conformational states. Specifically, the complex of gyrophoric acid with Aβ42 peptide exhibited relative stability after 70 ns, with RMSD values converging within a narrow range (1.2–1.8 Å), demonstrating that the system reached equilibrium in the latter phase of the simulation.

RMSF (root-mean-square fluctuation) analysis of the Aβ42 peptide chain revealed local conformational changes, with peaks corresponding to regions exhibiting the highest flexibility during the simulation. As shown in Figure 2b, the binding to gyrophoric acid increased structural flexibility in the protein, particularly within residue regions of 5–20AA, 20–30AA, and 45–55AA. Figure 2c depicts the interaction profiles between the ligand and key protein residues during the 100 ns simulation. The X-axis lists the specific binding site residues, while the Y-axis represents the fraction of simulation time at each interaction. While initial docking revealed no significant non-covalent interactions, subsequent MD simulations identifying protein–ligand interactions were monitored throughout the simulation. The interaction was categorized into four types: hydrogen bonds, hydrophobic contacts, ionic bridges, and water bridges. Hydrogen bonds are represented by green, hydrophobic interactions by purple, and water bridges by blue. Key residues critical for binding included Leu17 (chain A), Val40 (chain A), Ala42 (chain A), Leu17 (chain B), Leu17 (chain C), and Ala42 (chain E), with interactions dominated by water bridges, hydrophobic forces, and hydrogen bonds (Figure 2c). As a result, these residues underwent several interactions over the duration of simulation. Figure 2d shows frequent ligand contacts (indicated by darker orange shading) with residues Leu17 (chain A), Val40 (chain A), Ala42 (chain A), Leu17 (chain B), Leu17 (chain C), and Ala42 (chain E).

**Figure 3:**
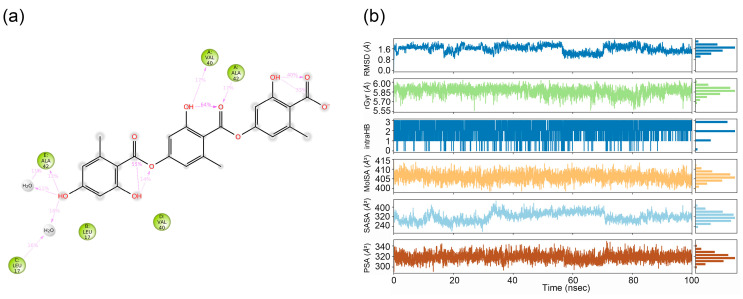
(**a**) Detailed schematic of the interactions between the compound and protein residues, showing interactions that occurred for more than 10.0% of the simulation time (i.e., interaction time exceeding 10 ns in a 100 ns simulation). Gyrophoric acid directly forms hydrogen bonds with chain A residue Ala42 (17%), chain A residue Val40 (17%), and chain E residue Ala42 (13%) while forming water-mediated bridges with chain E residue ALA42 (11%) and chain C residue Leu17 (16%). (**b**) Properties of the gyrophoric acid ligand during MD simulation. Ligand RMSD: root-mean-square deviation of a ligand with respect to the reference conformation (typically the first frame was used as the reference and regarded as time t = 0). Radius of gyration (rGyr): Measures the “extendedness” of a ligand and is equivalent to its principal moment of inertia. Intramolecular hydrogen bonds (intraHB): Number of internal hydrogen bonds (HB) within a ligand molecule. Molecular surface area (MolSA): Molecular surface calculation with 1.4 Å probe radius. This value is equivalent to the van der Waals surface area. Solvent-accessible surface area (SASA): Surface area of a molecule accessible by a water molecule. Polar Surface Area (PSA) is the portion of a molecule’s solvent-accessible surface area contributed solely by its oxygen and nitrogen atoms.

Figure 3 presents a schematic of detailed presents a schematic of detailed ligand–atom interactions with the protein residues that occurred more than 10.0% of the simulation time in the selected trajectory (0.00 through 100.01 ns) (Figure 3a). The diagram highlights interactions occurring for >10.0% of the simulation time (i.e., interaction durations exceeding 10 ns within the 100 ns trajectory). As shown in the left panel, gyrophoric acid directly forms hydrogen bonds with residues Ala42 (17%) (chain A), Val40 (17%) (chain A), and Ala42 (13%) (chain E) of Ab42 peptide. Water bridges mediated by intervening water molecules are observed with Ala42 (11%) (chain E), and Leu17 (16%) (chain C). Simulation time served as a quantitative metric to evaluate interaction stability and strength. Figure 3b presents six dynamic parameters of gyrophoric acid during the 100 ns MD simulation: RMSD stabilized near 1.6 Å (range: 0.8–1.8 Å), radius of gyration (rGyr) equilibrated at ~5.85 Å (5.55–6.00 Å), intense intramolecular hydrogen bonds occurred at 0–22 ns and ~87 ns, molecular surface area (MolSA) averaged 408 Å^2^ (395–415 Å^2^), solvent-accessible surface area (SASA) fluctuated widely (160–400 Å^2^), and polar surface area (PSA) ranged 280–340 Å^2^.

**Figure 4:**
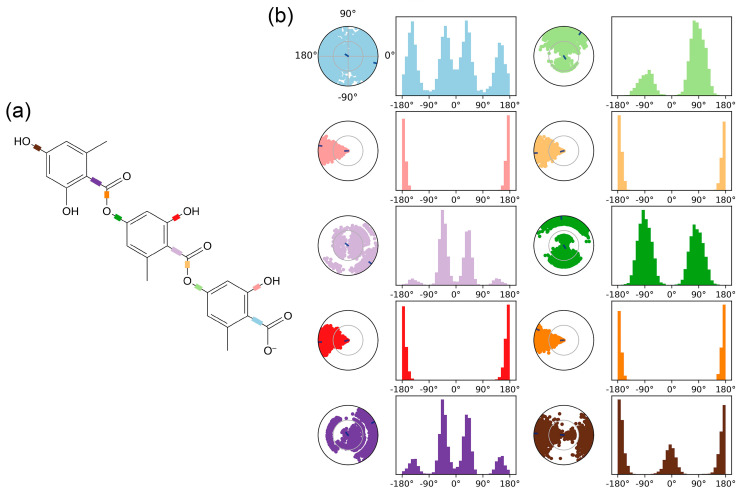
The ligand torsion plot summarizes the conformational evolution of each rotatable bond (RB) of the ligand (gyrophoric acid–Aβ) throughout the simulation trajectory. (**a**) 2D schematic of the ligand with color-coded RBs. Each RB’s torsion is accompanied by a dial plot and a bar graph in the same color. (**b**) Dial (or radial) plot illustrating the torsional conformations sampled during the entire simulation. The bar graph summarizes the data from the dial plot by showing the probability density of the torsion angles. (RB1: blue, RB2: pink, RB3: light purple, RB4: red, RB5: dark purple, RB6: light green, RB7: yellow, RB8: dark green, RB9: orange, RB10: brown).

Figure 4 comprehensively characterizes the conformational evolution of gyrophoric acid’s ten rotatable bonds (RBs) within the Aβ complex throughout the 100 ns simulation. Figure 4a shows a 2D schematic of the ligand with color-coded RBs. Each RB’s torsion (Figure 4b) is accompanied by a dial plot and a bar graph in the same color. The ligand torsion analysis of flexibility patterns: RB1 (blue), RB3 (light purple), and RB5 (dark purple) exhibit broad angular sampling (0–360°), while RB6 (light green) and RB8 (dark green) exhibit angular sampling (0–150° and 240–300°) with bimodal probability distributions, indicating multi-stable states. In contrast, RB2 (pink), RB4 (red), RB7 (yellow), and RB9 (orange) maintain rigid torsional confinement at 180° ± 30° (88% occupancy), while RB10 (brown) shows moderate flexibility within 180° ± 30° and 0° ± 60° ranges. This heterogeneity in rotational freedom—quantified through probability density histograms—correlates with steric constraints imposed by Aβ’s binding pocket, where rigid bonds (RB2/RB4/RB7/RB9) anchor the ligand core while flexible termini (RB3/RB5/RB6/RB8) facilitate adaptive interactions.

### 2.3. ADMET Properties and Druglikeness Prediction

A comprehensive understanding of pharmacology and toxicology is essential to advance drug development. Such knowledge can reduce development timelines and enhance success rates. ADMET is frequently used to evaluate the properties of a substance. ADMET simulations of gyrophoric acid revealed moderate oral absorption potential (PHOA: 48.5–57.3%) and favorable human serum albumin binding (QPlogKhsa: −0.69 to 0.35), aligning with pharmacokinetic safety thresholds (Table 1). The compound exhibited molecular weights (372.3–468.4 Da) and solvent-accessible surface areas (SASA: 525.8–763.1 Å^2^) within the druglike ranges. Gyrophoric acid displayed the highest polar surface area (PISA: 224.7 Å^2^) and molecular volume (1363.6 Å^3^), correlating with its larger structure. Despite suboptimal PHOA scores (<80%), the metabolites’ moderate absorption profiles combined with low risks of excessive albumin binding (QPlogKhsa within −1.5–1.5) suggest potential systemic bioavailability. These properties, paired with their previously reported Aβ peptide affinity, position gyrophoric acid as viable candidates for further optimization, particularly for peripheral therapeutic applications.

**Figure 5:**
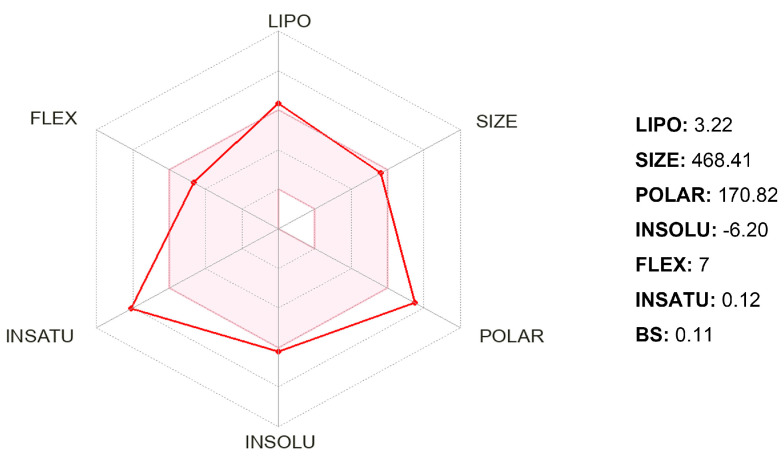
Bioavailability radar of gyrophoric acid using SwissADME. Pink area = most desirable area for each of the bioavailability properties: LIPO = lipophilicity, POLAR = polarity, INSOLU = insolubility, FLEX = flexibility, SIZE = molecular weight, INSATU = insaturation.

The bioavailability radar provided a quick overview of the relevant pharmacological characteristics and druglikeness of gyrophoric acid. In Figure 5, the pink regions indicate the most desirable area for each of the bioavailability properties (LIPO, SIZE, INSOLU, POLAR, INSATU, and FLEX) and indicate the optimum range for six properties: lipophilicity (XLOGP3 between −0.7 and +5.0), size (MW between 150 and 500 g/mol), polarity (TPSA between 20 and 130 Å^2^), solubility (log S not exceeding 6), saturation (fraction of carbons in sp3 hybridization not less than 0.25), and flexibility (no more than nine rotatable bonds). The red distorted hexagon within the pink region represents the druglikeness features. It was found that gyrophoric acid was slightly outside the pink area on one side due to the inconformity of saturation, polarity, lipophilicity, and solubility.

Druglikeness evaluates the molecular and structural similarity of compounds to known pharmaceuticals, which requires balanced assessment of hydrophobicity, electronic distribution, hydrogen bonding, molecular weight, pharmacophoric groups, bioavailability, reactivity, toxicity, and metabolic stability [27]. Lipinski’s rule of five (RO5) is widely used to predict absorption/permeation. Violations typically indicate suboptimal bioavailability. SwissADME analysis [28] confirmed that gyrophoric acid fully complied with RO5 parameters, suggesting favorable absorption, positioning the acid as a viable lead compound. Pharmacokinetic profiling further indicated a high gastrointestinal absorption. Additional filters, including PAINS structural alerts in identifying instability/toxicity risks [29,30] and synthetic accessibility (SA) scoring, were applied to assess the reactivity and synthetic feasibility.

### 2.4. Gyrophoric Acid Disassembles Aβ42 Fibrils

**Figure 6:**
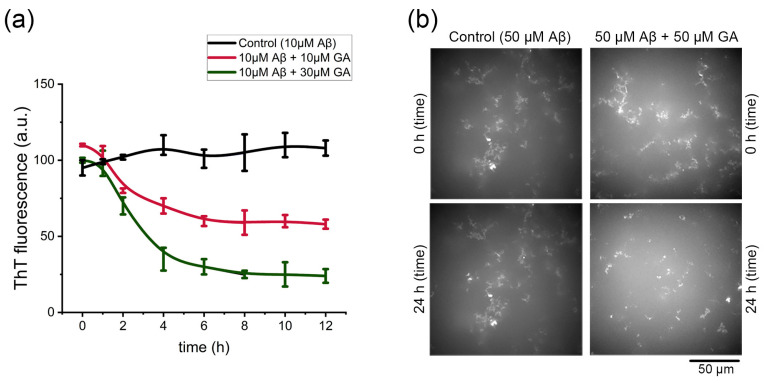
Disaggregation effect of gyrophoric acid (GA) against mature Aβ fibrils. (**a**) Final ThT fluorescence of mature Aβ42 fibrils after 3-day incubation ± GA (10–30 μM). Data are presented as means ± SD (*n* = 4 per group). (**b**) Confocal images of control (50 μM Aβ42) and gyrophoric acid-treated (50 μM Aβ42 + 50 μM GA) samples at 0 and 24 h.

The deposition of mature Aβ42 fibrils in the brain contributes to neuronal death, and the toxic oligomers are often generated through secondary nucleation catalyzed by existing fibrils [31]. To investigate whether gyrophoric acid disrupted these fibrils, we monitored Aβ42 aggregate structures using the assays of in vitro ThT fluorescence and confocal microscopy. ThT analysis (Figure 6a) revealed that the untreated Aβ42 fibrils remained stable over time. In contrast, the co-treatment with gyrophoric acid induced a dose-dependent disassembly of Aβ42 fibrils. This disassembly reached maximal efficacy within 5 h and remained stable thereafter.

To further characterize the disaggregation effect of gyrophoric acid in real time, we performed confocal microscopy on freshly dissolved Aβ42 fibrils incubated with or without gyrophoric acid. After 24 h in PBS at room temperature, the control fibrils (50 µM Aβ42) exhibited minimal structural change (Figure 6b, from Supplementary Video S1). Conversely, the fibrils co-treated with gyrophoric acid (50 µM Aβ42 + 50 µM) displayed significant disassembly compared to the initial state (0 h). Time-lapse imaging captured hourly from 0 to 24 h demonstrated that gyrophoric acid treatment markedly reduced the number of larger aggregates compared to the control, indicating dissolution (Supplementary Video S1).

## 3. Discussion

Our integrated analyses establish gyrophoric acid—a characteristic lichen depside [32]—as a dual-action inhibitor of Aβ42 aggregation, operating through distinct structural mechanisms. Thus, lichen-derived depsides represent a pharmacologically rich, yet underexplored class of natural products. Their rigid aromatic scaffolds and high phenolic content enable unique biomolecular interactions, providing exceptional surface complementarity with amyloidogenic targets that synthetic compounds often fail to achieve.

Computational studies, as described here, resolved gyrophoric acid’s paradoxical binding signature by revealing multiphase processes driven by entropic burial of hydrophobic side chains at key residues (Ala42, Val40, and Leu17). This conformational selection mechanism, validated through 100 ns molecular dynamic simulations, demonstrated a stable complex formation (RMSD plateau: 1.2–1.8 Å after 70 ns) and induced flexibility in nucleation-critical regions.

Complementing computational predictions and biochemical assays confirmed rapid disassembly of preformed fibrils (<5 h) triggered by gyrophoric acid, aligning with prior evidence of its neuroprotective effects in cell models [16]. Our earlier study identified superior Aβ inhibition by ethanol extracts of *Lobaria* containing gyrophoric acid; however, the study lacked the comprehensive in silico analysis presented here. Furthermore, the time-lapse confocal microscopy here quantitatively confirmed near-complete dissolution of large aggregates within 24 h.

A natural depside, gyrophoric acid exhibits favorable druglike properties consistent with the evolutionary advantages of lichen compounds. Biosynthesis under extreme environmental conditions confers remarkable metabolic stability and low cytotoxicity. ADMET profiling indicates low hepatotoxicity and minimal CYP450 inhibition, though these predictions carry inherent software limitations: QSAR models underpredict phase II metabolism of polyphenolic scaffolds and cannot capture tissue-specific toxicity mechanisms. However, the high polar surface area (224.7 Å^2^) suggests limited BBB permeability of gyrophoric acid. This challenge reflects a broader limitation for lichen-derived polyphenols, where high molecular weight often compromises BBB penetration. Nevertheless, the exceptional target stabilization (ΔG: −27.3 kcal/mol) and oxidative resilience offer compensatory advantages of these polyphenols. Computational ADME tools cannot quantify such target-mediated compensation, as they lack the coupled pharmacodynamic–pharmacokinetic modeling, ignore saturation effects from high-affinity binding, and overgeneralize clearance mechanisms for unique chemotypes.

Gyrophoric acid occurs in several lichen genera, e.g., *Umbilicaria*, *Lobaria*, *Parmotrema*, *Hypotrachyna*, and exhibits strong antimicrobial, antioxidant, and anticancer properties [14,15,16,33]. Traditional usage of lichen persists. In Himalayan regions, lichen is consumed as food and/or medicine. Growing domestic tourism has spurred market demand for the health-promoting lichen products, raising concerns about overharvesting due to low productivity. Concurrently, there is increasing interest in lichens as sources of innovative pharmacologically active compounds, particularly depsides and depsidones, e.g., gyrophoric acid, fumarprotocetraric acid, and lobaric acid, with known antibacterial, anti-inflammatory, and anti-cytotoxic properties [33]. Our innovative first-of-its-kind research methodology provides a new direction for studying lichen-derived natural products.

Positioned as a structurally novel hit compound, gyrophoric acid exemplifies the untapped potential of lichen symbionts in drug discovery for neurodegenerative diseases. Future work should prioritize: (i) in vivo validation; (ii) rational design of derivatives; and (iii) formulation strategies. Critically, lichen chemodiversity offers diverse scaffolds (depsidones, dibenzofurans) with modified ring topologies that could enhance BBB permeability while retaining anti-aggregation efficacy. Systematic screening of lichen metabolite libraries is needed to identify analogues with optimized pharmacokinetic profiles, leveraging co-evolutionary adaptations that confer both target specificity and environmental resilience.

## 4. Materials and Methods

### 4.1. Preparation of Aβ Fibrils

Synthetic Aβ42 powder (Sigma-Aldrich, St. Louis, MO, USA) was dissolved in 100% hexafluoro-isopropanol (HFIP) at 1 mM to disrupt preexisting aggregates. After sonication (20 min, 25 °C), the monomer solution was aliquoted, dried overnight in a fume hood to evaporate HFIP, and stored at −20 °C. For fibril formation, the peptide film was resuspended in 20 μL DMSO and diluted in 10 mM HCl to 100 μM Aβ42. The solution was incubated at 37 °C for 6 days to generate mature fibrils.

### 4.2. Thioflavin T (ThT) Fluorescence Assay

A ThT fluorescence assay was generated following the method described in a previous report [15]. Briefly, Aβ42 fibrils (10 μM) aged for 6 days were incubated with or without gyrophoric acid at 37 °C for 5 days. The ThT fluorescence was measured every two hours for 12 h in a 96-well black plate at excitation and emission wavelengths of 435 and 488 nm, respectively.

### 4.3. Confocal Microscopy Real-Time Measurement

Disaggregation kinetics were monitored using an Olympus IX73 confocal microscope (Tokyo, Japan) with a 60× objective. Samples (200 μL) included control (50 μM Aβ42 fibrils) and gyrophoric acid treatment (50 μM Aβ42 fibrils + 50 μM gyrophoric acid) in PBS. Samples were deposited on glass-bottomed dishes (10 mm diameter) and imaged hourly for 24 h at room temperature. Images were acquired with Micro-Manager software (ImageJ software, https://imagej.net/, accessed on 20 June 2024) and processed for quantitative analysis.

### 4.4. In Silico Analysis

The crystal structure of Aβ42 was retrieved from the largest structure repository, Protein Data Bank (PDB), with the PDB ID 2BEG (https://www.rcsb.org/structure/2BEG, accessed on 1 January 2025). To confirm the structural reliability of 2BEG crystal, the crystal structure was optimized using Protein Preparation Wizard module in Schrödinger software (Maestro 14.3 version) through the following sequential steps: protein preprocessing, regeneration of native ligand states, hydrogen bond assignment optimization, protein energy minimization, and water removal. Subsequent quality assessment was performed using the Ramachandran Plot module in Maestro 14.3 version software to evaluate the stereochemical reliability of the refined protein structure.

The obtained protein structure was preprocessed using the Protein Preparation Wizard module in Schrödinger Suite. This included the following steps: structural optimization (e.g., correcting bond orders, adding missing hydrogen atoms); regeneration of the native ligand’s protonation states at pH 7.4; hydrogen bond assignment optimization; energy minimization of the protein structure using the OPLS4 force field; and removal of crystallographic water molecules unrelated to binding. The 2D SDF structure file of the compound (gyrophoric acid) was processed using the LigPrep module in Schrödinger to generate all possible 3D chiral conformations. Ionization states were predicted at pH 7.4 ± 2.0, and low-energy stereoisomers were retained for subsequent docking studies. In active site identification, the SiteMap (Schrödinger) was employed to predict potential binding pockets on the Aβ42 peptide. The Receptor Grid Generation module was used to define an enclosing box around the top-ranked binding site identified by SiteMap, ensuring complete coverage of the predicted active region. In molecular docking, the prepared ligand (i.e., gyrophoric acid) was docked into the active site of the Aβ42 peptide using the Glide module in XP (extra precision) docking mode (Maestro 14.3 version). By highlighting the active site amino acid residues, grid box dimensions of the 2BEG receptor were generated in x, y, and z axes, which were −14.56, −4.08, and −9.04, respectively, with grid size of 10 Å.

### 4.5. MM–GBSA Binding Free Energy Calculations

The binding interactions between gyrophoric acid and the Aβ42 peptide were further evaluated using MM–GBSA (molecular mechanics–generalized Born surface area) calculations. The Prime MM–GBSA module in Schrödinger was utilized to estimate the binding free energy (ΔG_bind_), where lower ΔG_bind_ values correlated with enhanced ligand–protein binding stability.

### 4.6. MD Simulation

Molecular dynamic (MD) simulations were performed using Desmond (OPLS4 force field) to optimize compound–protein binding. The protein–ligand complex was solvated in a cubic water box (SPC/E model), neutralized with 0.15 M NaCl, and energy-minimized via steepest descent (50,000 steps). Systems underwent restrained NVT/NPT equilibration (50,000 steps; 300 K, 1 bar), followed by unrestrained 100 ns production runs. Trajectory analysis was conducted in Maestro 13.5.

### 4.7. ADMET and Bioavailability Radar Analysis

The drug discovery process necessitates early prediction of the ADMET of the potential pharmacological agents. In this study, ADMET analysis was conducted in Maestro 13.5. The two primary pharmacokinetic features, crucial for assessment of drug candidates for drug development, are blood–brain barrier (BBB) access and gastrointestinal tract absorption. The BOILED-Egg predictive model calculates the lipophilicity and the polarity of small compounds to determine their bioavailability. This prediction was carried out using SwissADME (http://www.swissadme.ch, accessed on 20 August 2025) in which gyrophoric acid was subjected to lipophilicity (WLOGP) and polarity (TPSA) computation in SMILES notation [34].

## 5. Conclusions

This study establishes gyrophoric acid as a dual-action anti-amyloid agent capable of disassembling mature fibrils through a unique hydrophobic-driven mechanism. Computational resolution of its binding paradox (weak docking affinity vs. strong MM–GBSA stabilization [−27.3 kcal/mol]) revealed entropic burial at key residues (Ala42, Val40, and Leu17). This was further validated by MD simulations demonstrating complex stability (RMSD: 1.2–1.8 Å) and enhanced fibril flexibility. Experimentally, gyrophoric acid showed rapid fibril dissolution (<5 h, ThT/confocal microscopy) and dose-dependent efficacy. Gyrophoric acid exhibits favorable ADMET properties, druglikeness, and complies with Lipinski’s rules [35]. The integration of computational and experimental approaches is essential to advance gyrophoric acid as a potential anti-Alzheimer’s therapeutic, paving the way for novel strategies against this disease.

## Figures and Tables

**Table 1:** ADMET simulation properties of gyrophoric acid.

Property or Descriptor with Range or Recommended Values	Gyrophoric Acid	Property or Descriptor with Range or Recommended Values	Gyrophoric Acid
Mol. Wt (130–725)	468.416	QPlogPo/w (−2.0–6.5)	3.256
SASA (300–1000)	763.078	QPlogS (−6.5–0.5)	−6.041
FOSA (0–750)	208.919	CIQPlogS (−6.5–0.5)	−6.959
FISA (7–330)	329.438	QPlogHERG (concern below −5)	−4.367
PISA (0–450)	224.721	QPlogBB (−3.0–1.2)	−3.595
Volume (500–2000)	1363.561	QPlogKp (−8.0–−1.0)	−5.935
donorHB (0.0–6.0)	2.000	IP(eV) (7.9–10.5)	9.524
accptHB (2.0–20.0)	7.000	EA(eV) (−0.9–1.7)	0.621
dip^2/V (0.0–0.13)	0.130	#metab (1–8)	7
ACxDN^.5/SA (0.0–0.05)	0.013	QPlogKhsa (−1.5–1.5)	0.354
glob (0.75–0.95)	0.780	HumanOralAbsorption	1
QPpolrz (13.0–70.0)	44.783	PHOA (<25% is poor, >80% is high)	50.942
QPlogPC16 (4.0–18.0)	15.378	QPlogKhsa (−1.5 to 1.5)	0.354
QPlogPoct (8.0–35.0)	23.996	RuleOfFive (maximum is 4)	0
QPlogPw (4.0–45.0)	13.164		

## Data Availability

The datasets generated during and/or analyzed during the current study are available from the corresponding author on reasonable request.

## Supplementary Materials

The following supporting information can be downloaded at https://www.mdpi.com/article/10.3390/ijms26178500/s1.

## Abbreviations

The following abbreviations are used in this manuscript.

AD	Alzheimer’s disease
Aβ	amyloid beta
ThT	thioflavin T
MD	molecular dynamics
ADMET	absorption, distribution, metabolism, excretion, toxicity
AChE	acetylcholinesterase
MM–GBSA	molecular mechanics–generalized Born surface area
RMSD	root-mean-square deviation
