# Supplementary material for: The Identification of Gyrophoric Acid, a Phytochemical Derived from Lichen, as a Potent Inhibitor for Aggregation of Amyloid Beta Peptide: In Silico and Biochemical Evaluation
Source: Int J Mol Sci. 2025 Sep 1;26(17):8500. doi: 10.3390/ijms26178500 (PMC12428957; doi:10.3390/ijms26178500)
Supplement: Supplementary file 1 [file ijms-26-08500-s001.zip › Suppl Fig Legends.pdf]

Supplementary Figure Legends

Supplementary Figure S1. The Ramachandran plot assesses the distribution of amino acid residues in the Aβ42 structure (PDB ID: 2BEG) across stereochemically defined regions. A structure is considered stereochemically valid when >90% of residues reside in the *allowed* (red) and *marginally allowed* (yellow) regions. An elevated number of residues in disallowed regions (white areas, marked as red dots) suggests potential model inaccuracies. For 2BEG, fewer than 10% of residues occupy disallowed regions, confirming high structural reliability. These outlier residues are predominantly located in flexible segments of the protein and do not significantly impact molecular docking.

Supplementary Figure S2. 2D **(a)** and 3D **(b)** diagrams of curcumin docked with the amyloid beta peptide protein. Curcumin binds to the surface of the active pocket of amyloid beta peptide, forming hydrophobic interactions with residues VAL39 and VAL40 on Chain E, VAL40 and LEU17 on Chain C, and LEU17 on Chain B. Additionally, curcumin forms π-π stacking interactions with PHE19 on Chain D and PHE19 on Chain E.
