# Supplementary material for: The Identification of Gyrophoric Acid, a Phytochemical Derived from Lichen, as a Potent Inhibitor for Aggregation of Amyloid Beta Peptide: In Silico and Biochemical Evaluation
Source: Int J Mol Sci. 2025 Sep 1;26(17):8500. doi: 10.3390/ijms26178500 (PMC12428957; doi:10.3390/ijms26178500)
Supplement: Supplementary file 1 [file ijms-26-08500-s001.zip › Suppl Table S1-MM-GBSA Binding Free Energy Calculations.pdf]

Supplementary Table S1. MMGBSA dG Bind results of curcumin and gyrophoric acid, the binding free energy between the ligand and receptor (Amyloid beta peptide).

**Title**	**Curcumin**	**Gyrophoric acid**
PUBCHEM_MOLECULAR_FORMULA	C21H20O6	C24H20O10
XP GScore	-7.455	-1.121
**MMGBSA dG Bind**	**-30.57**	**-27.3**
MMGBSA dG Bind Coulomb	1.77	29.48
MMGBSA dG Bind Covalent	4.37	2.56
MMGBSA dG Bind Hbond	-0.04	-0.07
MMGBSA dG Bind Lipo	-32.52	-15.06
MMGBSA dG Bind Packing	-1.99	0
MMGBSA dG Bind SelfCont	0	0
MMGBSA dG Bind Solv GB	13.78	-15.29
MMGBSA dG Bind vdW	-15.94	-28.92

MMGBSA dG Bind = MMGBSA dG Bind (Coulomb + Covalent + Hbond + Lipo + Packing + SelfCont + Solv GB + vdW)
