# Supplementary figures and images for: The Identification of Gyrophoric Acid, a Phytochemical Derived from Lichen, as a Potent Inhibitor for Aggregation of Amyloid Beta Peptide: In Silico and Biochemical Evaluation
Source: Int J Mol Sci. 2025 Sep 1;26(17):8500. doi: 10.3390/ijms26178500 (PMC12428957; doi:10.3390/ijms26178500)

Suppl Fig. S1

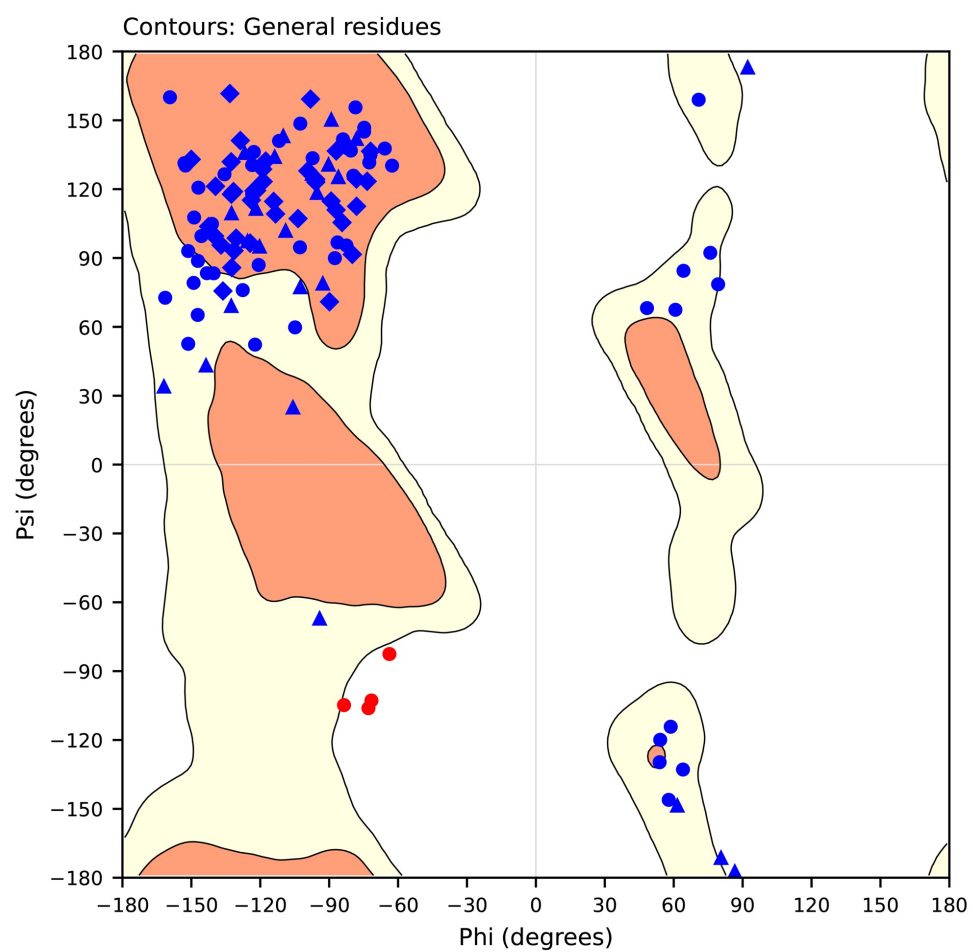

Supplement: Supplementary file 1 [file ijms-26-08500-s001.zip › Suppl Fig. S1-Ramachandran plot analysis .pdf]

## Suppl Fig. S2

(a)

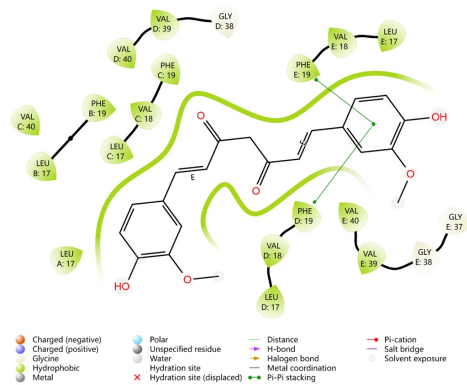

(b)

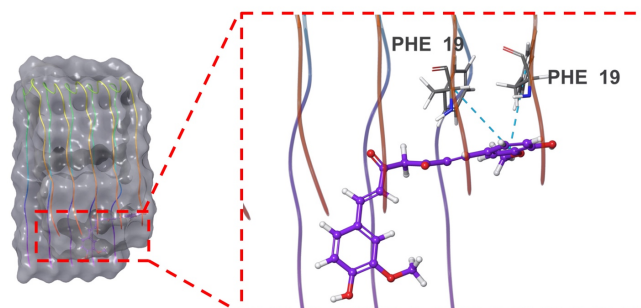

Supplement: Supplementary file 1 [file ijms-26-08500-s001.zip › Suppl Fig. S2-XP molecular docking of amyloid beta peptide with curcumin.pdf]
